# Vaccination Recommendations for the Hematology and Oncology and Post–Stem Cell Transplant Populations

**DOI:** 10.6004/jadpro.2012.3.2.2

**Published:** 2012-03-01

**Authors:** Vivian Tsang

**Affiliations:** From Cedars-Sinai Medical Center, Los Angeles, California

## Abstract

Vaccination is a simple yet important process used to prevent many infections in the general population. For patients with suppressed immune systems, especially those who are undergoing chemotherapy or who have undergone stem cell transplant, repeat vaccination or boosters may be crucial in prolonging and/or extending immunity. The purpose of this review is to examine the need for each vaccine in two separate oncology populations: patients receiving concurrent chemotherapy and post–stem cell transplant patients. In addition, the importance of avoiding live vaccines and criteria for reconsideration at a future time will also be discussed.

Despite the implementation of appropriate vaccinations for a person with a healthy immune system, immunity is not always achieved. For example, some individuals never establish immunity after receiving a hepatitis B vaccine (HBV) series (Egea et al., 1991; Wood et al., 1993). Similarly, oncology patients who have received chemotherapy or hematopoietic stem cell transplantation (HCST) may have poor uptake or may be unable to mount an effective immune response postvaccination. In order to accurately assess a patient’s response to a specific vaccination, it is suggested that a baseline titer be drawn prior to treatment and compared to posttreatment levels (Ring et al., 2003).

For a healthy immune system, it typically takes up to 2 weeks after vaccination for the adaptive immunity to respond to the exposed pathogen. In the oncology population, concurrent chemotherapy and immune reconstitution posttransplant are two factors that may alter the effectiveness of vaccinations as well as the recovery process of the immune system. As a result, the timing of vaccinations with respect to treatment may play a role in achieving extended immunity and better outcomes for oncology patients (Pollyea et al., 2010).

The Centers for Disease Control and Prevention (CDC) have established guidelines detailing recommended routine vaccination schedules for various populations. For healthy individuals, the recommended schedules for the different age groups are available through the CDC website (CDC, 2012). While these guidelines also include high-risk patients, the timing and specific recommendations for the oncology population are inadequate. This review will focus on the need for appropriate timing of specific vaccinations in two adult oncology populations: those who are receiving chemotherapy and those who have undergone stem cell transplantation.

## Immunity to Vaccine-Preventable Diseases

While infection remains the leading cause of posttransplant complications, protection against vaccine-preventable infections remains a priority. Many patients have undergone childhood vaccination per the CDC guidelines. As an adult, the need for boosters is recommended based on a recent outbreak or the demonstration of a decrease or loss in immunity. In patients undergoing transplant, the loss of pretransplant immunity is inevitable. The degree of immunity loss may be dependent on several factors such as the strength of the existing immunity, the type of transplant, the source of the stem cells, the conditioning regimen used, the presence and severity of graft-vs.-host disease (GVHD), and the immunosuppression used (Ljungman et al., 2005).

Following the suppression of the immune system, the body’s natural course of recovery (otherwise known as immune reconstitution) begins at the blood cell line level, followed by B-cell recovery, and finally T-cell recovery. After high-dose cytotoxic therapy, once nadir is reached, blood cell line recovery begins at 2 to 4 weeks followed by B- and T-cell recovery at approximately 1 to 3 months posttransplant. As a result of the delayed recovery, a fully functional immune system is not obtained until approximately 6 to 12 months posttransplant (Singhal & Mehta, 1999). Despite eventual recovery of the immune system, some posttransplant patients are viewed as "never vaccinated," thereby requiring specific reimmunization for certain vaccines while avoiding others.

## Influenza Vaccine

According to the CDC, an estimated 5% to 20% of the general population is affected by influenza each year. Despite the availability of vaccines, influenza still accounts for over 200,000 hospitalizations and roughly 35,000 deaths each year (> 90% in older adults) (Thompson et al., 2003 & 2004). Influenza A and B are two subtypes responsible for this viral illness. Symptoms of influenza may include fever and myalgia, with or without lower respiratory tract symptoms. Influenza A is further defined based on surface antigens (hemagglutinin and neuraminidase), and influenza B by genetic lineages. Each year, the World Health Organization (WHO) and the CDC produce influenza vaccine targeting specific anticipated strains.

In the general oncology population, the low vaccination rate and insufficient immunologic response to the influenza vaccine are two contributing factors to the high influenza mortality rate: up to 90% (Loulergue et al., 2008) vs. < 10% in the nononcology population (CDC, 2012). In adults, the ability to mount a protective response to the influenza vaccine was observed in 17% to 76% of patients with hematologic malignancies and 41% to 83% of patients with solid tumors (Table 1).

**Table 1:**
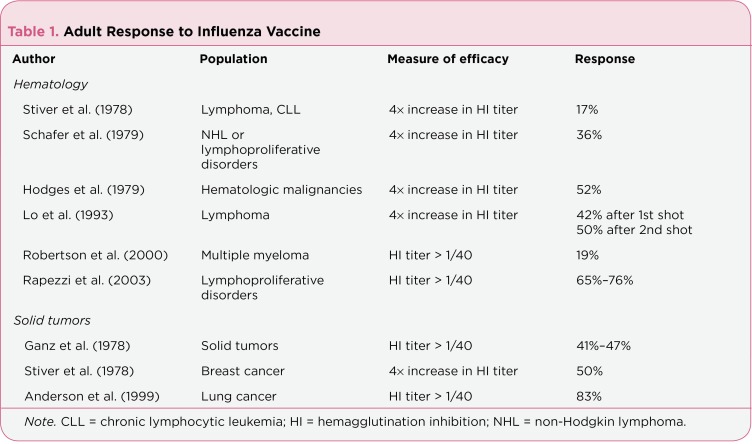
Table 1. Adult Response to Influenza Vaccine

With a general 2-week lag time for an antibody response postvaccination, it is recommended that at-risk patients obtain the influenza vaccine prior to the peak of the season. Per the CDC, annual vaccination against influenza is recommended (CDC, 2012). Patients should be vaccinated at least 2 weeks before starting chemotherapy or in between cycles, with the former scenario likely more beneficial (Sommer et al., 2006; Melcher, 2005).

In the HSCT population, it is recommended that vaccination with the inactivated influenza vaccine should take place no sooner than 6 months posttransplant but within the first year after transplant. A second dose, 1 month later, should be considered, especially if it is the first time the patient has ever been exposed to the influenza vaccine. Due to a compromised immune system posttransplant, live attenuated influenza is contraindicated in this population. It is also recommended that family members and caretakers also receive annual inactivated influenza vaccination if there is contact with those who are immunosuppressed (Ljungman et al., 2009).

The observed side effects of the inactivated influenza vaccine are similar in all populations in that they are generally mild and may include soreness or pain around the injection site, fever, fatigue, or myalgia. These symptoms may occur 6 to 12 hours postinjection and generally last no longer than 2 days. In a rare case scenario Guillain-Barré Syndrome, a neurologic complication, may occur (Shoji & Kaji, 2003).

The two inactivated influenza vaccine types and dosing recommendations are presented in Table 2. Patients with a known allergic reaction or hypersensitivity to eggs should not receive the influenza vaccine. While it is not contraindicated to vaccinate patients who present with neutropenia, timing of the influenza vaccination with respect to their acute or chronic conditions should be considered.

**Table 2:**
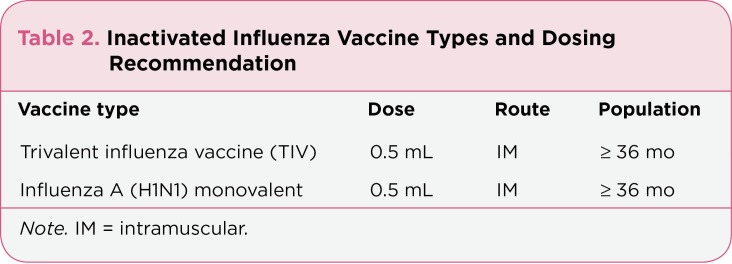
Table 2. Inactivated Influenza Vaccine Types and Dosing Recommendation

## Pneumococcal Vaccine

The National Foundation for Infectious Diseases (NFID) reports that an estimated 175,000 hospitalizations each year are attributed to pneumococcal pneumonia. *Streptococcus pneumonia* and 90 other strains of pneumococcus are the primary culprits of such bacterial infections affecting many parts of the body. When this infection invades the lungs, blood, and brain, it results in pneumonia, bacteremia, and meningitis, respectively. While the incidence of severe pneumococcal infection accounts for a small portion of all pneumococcal infections, the mortality rate remains at approximately 7% (NFID, 2011). If appropriate vaccination is recommended and instituted for at-risk patients, it would likely have a significant impact on the improvement of mortality. The pneumococcal vaccine is generally recommended for very young and very old individuals, and particularly for those who are immunosuppressed and asplenic.

Currently, two types of pneumococcal vaccines are available. Pneumococcal conjugate vaccine (PCV7) and pneumococcal polysaccharide 23-valent vaccine (PPSV23) are recommended for pediatric and adult populations, respectively. In the general pediatric population, PCV7 serotype specific efficacy could reach upwards of 85% (Black et al., 2000). PPSV23 has an overall effectiveness of 50% to 70% in preventing pneumococcal bacteremia in the adult population (Mangtani, Cutts, & Hall, 2003; Fedson, 1999; Fine et al., 1994), but fails in the prevention of nonbacteremia pneumonia or otitis media (Table 3). Due to the variation in immunogenicity between PCV7 and PPSV23, there are different recommendations for these pneumococcal vaccines.

**Table 3:**
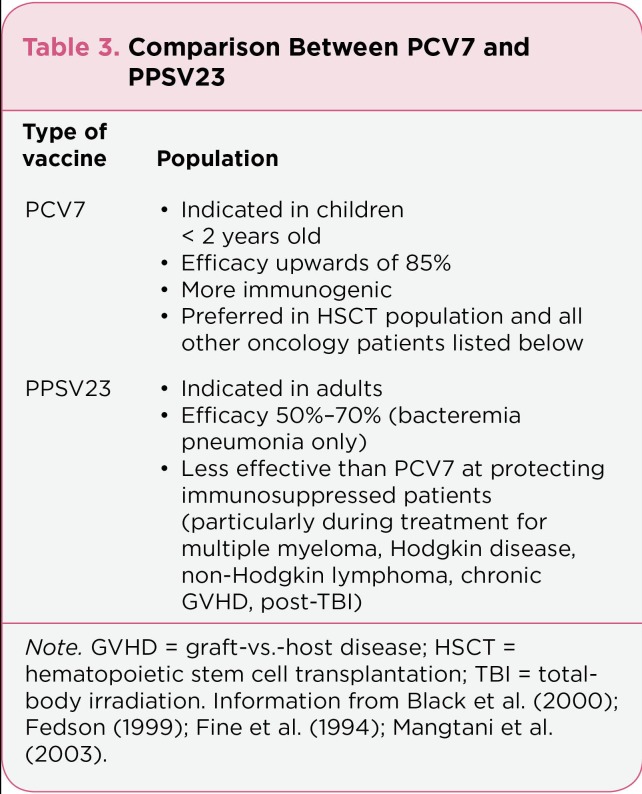
Table 3. Comparison Between PCV7 and PPSV23

In general, adult patients who are receiving chemotherapy treatment for both hematologic (with some exceptions) and oncologic conditions should receive the pneumococcal vaccine (PPSV23) 4 to 6 weeks (minimum 2 weeks) prior to starting chemotherapy; revaccination should occur once 5 years after the initial dose, then as recommended by vaccination guidelines (CDC, 2012; Arrowood, 2002). Due to a lower immunogenic response, patients receiving treatment for multiple myeloma, Hodgkin and non-Hodgkin lymphomas, and chronic graft-vs.-host disease (cGVHD), as well as those who have undergone total-body irradiation, should be vaccinated with PCV7 instead of PPSV23 (Mangtani, Cutts, & Hall, 2003; Fedson, 1999; Fine et al., 1994).

Approximately 1% to 10% of the HSCT population will contract a serious pneumococcal infection, with a median onset of 1 year posttransplant (Engelhard et al., 2002; Kulkarni et al., 2000). Timing and vaccine formulation are critical for maximizing the efficacy of pneumococcal vaccines. In four prospective trials, HSCT recipients obtained better response from PCV7 as compared to PPSV23, perhaps because PCV7 is more immunogenic. For this reason, PCV7 is the preferred agent, with a three-dose series followed by one dose of PPSV23 to broaden the immune response (Cordonnier et al., 2008; Kumar et al., 2007; Meisel et al., 2007; Molrine et al., 2003). Patients should begin receiving PCV7 at 6 months posttransplant with 1-month intervals between each of the first three doses, then a PPSV23 booster at 18 months after transplant to maximize the duration of the pneumococcal protection (Hilgendorf et al., 2011). If pneumococcal vaccination took place early posttransplant before the 6-month mark, the duration of antibody response could be suboptimal; therefore, antibody titer should be determined and revaccination implemented as deemed appropriate (Hilgendorf et al., 2011; Ljungman et al., 2009).

Common adverse reactions related to the pneumococcal vaccines are soreness at the injection site and fever. Severe reactions are rare. Patients with an acute infection—particular oncology patients who are undergoing or have recently undergone chemotherapy—should have pneumococcal vaccination postponed until resolution of the infection.

## Diphtheria-Pertussis-Tetanus Vaccines

Diphtheria is a transmissible upper respiratory tract infection caused by *Corynebacterium diphtheriae.* It spreads through direct contact with aerosolized and infected secretions. Since the implementation of vaccination programs, there have been fewer cases of tetanus and diphtheria cases each year in the United States. Pertussis, also known as whooping cough, is a highly contagious bacterial infection caused by *Bordetella pertussis.* Due to the potential for recurrence epidemiologically, an estimated 48 million cases and 295,000 deaths are attributed to pertussis worldwide (Bettiol et al., 2010). Tetanus, or lockjaw, is a preventable disease that affects the muscles and nerves caused by *Clostridium tetani.* The usual path of entry is a break in the skin. In all three instances, the primary method of prevention for tetanus, diphtheria, and pertussis is vaccination.

In order to lessen the discomfort of receiving several vaccines at once, a series of combination vaccines have been made available. Currently, there are several combination products available as a single injection containing tetanus and diphtheria vaccines with or without pertussis vaccines: Td, Tdap, and DTap. In general, the abbreviation DTP is used to refer to the triple combination products on the whole. The diphtheria toxoid component further defines the differences between these products. The lower case "d" in Tdap represents a reduced dose of diphtheria toxoid as compared to the upper case "D" in DTap, which contains a full dose of diphtheria toxoid. The tetanus component is the same in Td, Tdap, and DTap, while acellular pertussis is included in the latter two products only.

For patients with hematologic and oncologic malignancies, Td is recommended once every 10 years; this is the same as the recommendation for the general public (CDC, 2012). It is extremely important for patients who are receiving chemotherapy to be updated with the tetanus-diphtheria vaccination (Arrowood & Hayney, 2002). Due to the recent outbreak of pertussis, it is also recommended that a single dose of Tdap replace one of the routine Td boosters. DTap is approved for individuals less than 7 years old. Tdap is recommended for healthy adults who have received full-dose diphtheria as a child in order to avoid the potential for a local reaction from repeated exposure to full-dose diphtheria (CDC, 2012).

In contrast to their oncology and hematology counterparts, posttransplant patients are poor responders to Tdap. It is recommended that posttransplant patients receive DTap due to its higher response rate as compared to Tdap (Ljungman et al., 2009; Hilgendorf et al., 2011). Post-HSCT patients are viewed as "never vaccinated" and are at a lower risk of local reaction from DTap than healthy individuals. As such, DTap may be more immunogenic and should be considered the initial vaccination posttransplant. Patients should receive the DTap three-dose series beginning 6 months posttransplant in monthly intervals, followed by a booster 18 months after HSCT (Hilgendorf et al., 2011).

As with all other vaccines, the common adverse events include local injection site reaction and fever. Additional adverse events may include drowsiness and anorexia, which may be self-limiting. Rare but severe adverse events may include Guillain-Barré syndrome, neurologic disorders, uncontrollable seizures, prolonged convulsions, or acute encephalopathy. These serious and acute neurologic symptoms generally occur within the first 3 days after administration of DTP as compared to Td.

## *Haemophilus influenzae* Type B Conjugate Vaccine 

Historically, *Haemophilus influenzae* was thought to be the primary cause of influenzae. In the 1930s, the distinction was made between bacterial-induced *H. influenzae* and viral-induced influenzae. *H. influenzae* is often presented as a respiratory tract infection, but may rarely cause bacteremia, pneumonia, and meningitis. Since the implementation of the Hib conjugate vaccine in the 1990s, the prevalence of invasive disease has dramatically decreased by 99% to less than 1 per 100,000 cases (CDC, 2008).

In hematologic and oncologic adult patients, the need for a vaccine booster is based on antibody titer. It has been demonstrated that in patients with Hodgkin disease and leukemia, posttreatment antibody response to the Hib vaccine is suppressed as compared to pretreatment levels. In addition, antibody titers used to confer protection are lower in cancer patients as compared to the general population. In order to ensure optimal protection against *H. influenzae*, particularly for this population (Hodgkin disease and leukemic patients), patients should have titers tested both pre- and posttreatment; revaccination may be considered if the titers remain low posttreatment (Pirofski & Casadevall, 1998). If the titer is low, revaccination should be administered at least 2 weeks prior to starting chemotherapy to ensure maximal response or at least 3 months after completion of chemotherapy (Arrowood & Hayney, 2002).

In the HSCT population, a three-dose series beginning 6 months posttransplant, followed by a booster at 18 months posttransplant, is recommended (Hilgendorf et al., 2011; Ljungman et al., 2009). The response to postvaccination antibody level is augmented if HSCT recipients received donor-immunized hematopoietic stem cells as opposed to nonimmunized stem cells (Molrine et al., 1996). As such, it is extremely important that the donor receive up-to-date vaccination prior to stem cell collection while the recipient receives a series of appropriate vaccinations posttransplant.

Common adverse events associated with the Hib vaccine may include pain and swelling at the site of the injection. Other adverse events may include fever, drowsiness, anorexia, and vomiting.

## Hepatitis B Vaccine

Hepatitis B virus (HBV)–related infection primarily arises through the transmission of bodily fluids. While its incidence remains low, patients undergoing chemotherapy with or without HSCT may have the potential to contract HBV infection through blood transfusions. But in today’s practice, blood products routinely undergo a series of rigorous testing for the detection of potential infection that could be transferred to the recipient. At the instance when the patient is most vulnerable—when the immune system is suppressed— the likelihood of contracting an acute HBV infection, as well as the potential for reactivating chronic liver infections, is increased. Table 4 reflects the different stages of infection based on the various hepatitis parameters.

**Table 4:**
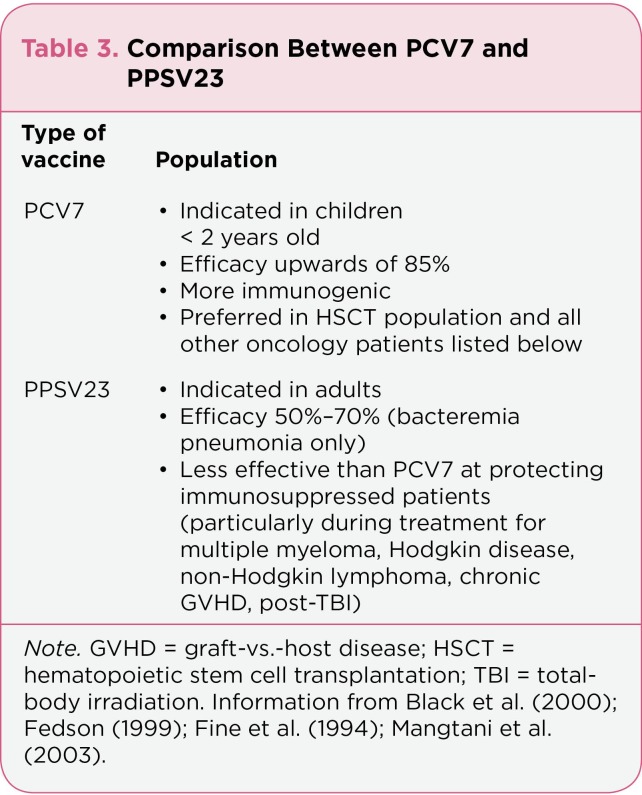
Table 4. Hepatitis B Markers and Various Stages of Infection and Vaccination

Hepatitis B virus vaccine is not required for previously vaccinated patients undergoing chemotherapy alone, as immunogenicity is sustained throughout treatment, unless hepatitis B antigen is undetectable by titer (Arrowood & Hayney, 2002). In a study of patients who had not previously been exposed to hepatitis B, or who had an undetectable titer level, and received the HBV vaccine prior to chemotherapy, 70% achieved adequate antibody response during the initial 12-month observational period posttreatment. This immunogenicity to the HBV vaccine was associated with increased survival rate (Weitberg et al., 1985). This demonstrated safe and effective ability of hepatitis B vaccine in inducing immunity in patients undergoing chemotherapy who have not previously been vaccinated. In post-HSCT patients, there are different recommendations based on hepatitis B history (Table 5).

**Table 5:**
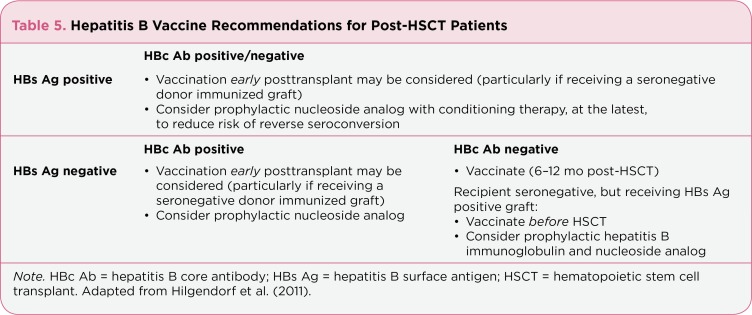
Table 5. Hepatitis B Vaccine Recommendations for Post-HSCT Patients

Patients who were previously HBs Ag positive or HBc Ab positive alone or HBs Ag positive *and* HBc Ab positive have the potential to reactivate the hepatitis B virus, particularly while immunosuppressed or immediately posttransplant (Hilgendorf et al., 2011). With the different combinations of antigen or antibody positivity, the recommendation and timing for hepatitis B vaccination differs. It is also important to note that early vaccination while receiving a donor-immunized graft helps augment the transfer of donor immunity in at least 50% of patients (Ljungman et al., 2005).

Specific patients without antibodies to hepatitis B or not at risk of reactivation prior to transplant may delay their hepatitis B vaccination until 6 to 12 months posttransplant as this population is not at immediate risk. In general, hepatitis B vaccine can reduce the risk of reverse seroconversion in patients with a history of hepatitis B infection. In patients who have resolved hepatitis B, or HBc Ab positive alone prior to transplant, prophylactic nucleoside analog should be initiated with the start of conditioning therapy at the latest. In addition, the response to early posttransplant vaccination with hepatitis B may be augmented if the donor is immunized prior to stem cell collection (Ljungman et al., 2005; Hilgendorf et al., 2011). Despite being seronegative recipients at baseline, the population at greatest risk is those patients who will be receiving a HBs Ag (positive) graft. In this population, hepatitis B vaccination before transplant, as well as the initiation of prophylactic hepatitis B immune globulin and nucleoside analog, is recommended (Hilgendorf et al., 2011).

Even with vaccination before transplant, posttransplant vaccination will need to be instituted to ensure long-lasting immunity (Ilan, 2000). For this reason, posttransplant patients should receive the three-dose series of hepatitis B vaccine at 1-month intervals, followed by a booster at 18 months post-HSCT (Hilgendorf et al., 2011).

Common adverse events related to hepatitis B vaccine include fever and pain at the injection site. Anaphylaxis and hypersensitivity are rare but serious adverse events that have been associated with the hepatitis B vaccine.

## Hepatitis A Vaccine

Ingestion of contaminated food or water is the usual route for hepatitis A infection. Direct contact with an infected individual could also lead to acute infection. Although it is self-limiting in most cases, rare but serious acute liver failure can occur. The incidence of hepatitis A infection is highest in underdeveloped countries and in areas of (or individuals with) poor hygiene practice. Vaccination plays a major role in preventing hepatitis A infection; its protective effects can last for more than 20 years. In healthy patients, two doses of hepatitis A vaccine will induce protection in up to 99% of recipients (CDC, 1999).

Unless one is traveling to endemic areas or requires postexposure prophylaxis, hepatitis A vaccine is optional. In general, two doses of monovalent hepatitis A vaccine given 6 months apart may be recommended. In posttransplant patients, hepatitis A vaccine recommendations are the same as for the general population. Common adverse events may include mild fever, headache, and pain at the injection site. Patients with prior risk of anaphylactic reaction to this vaccine should not receive any further doses.

## Polio Vaccine 

Poliomyelitis (polio) is an acute viral infection that is spread by direct contact via the fecal-oral route of entry. Infantile paralysis is another term that has been used to refer to polio, as it caused detrimental paralysis during the infant years in up to 20,000 cases before the introduction of the polio vaccine. As the infection attacks the human body, it produces inflammation along the spinal cord, causing muscle weakness and paralysis. Developed in the 1950s, inactivated poliovirus vaccine (IPV) was the first polio vaccine intended to protect individuals against polio. Through various manipulations, an enhanced-potency IPV was licensed in 1987 as the vaccine of choice against polio. Oral polio vaccine (OPV) is a live-attenuated vaccine that has excellent GI immunity compared to IPV (Pirofski, 1998).

In patients with hematologic and oncologic malignancies, revaccination with IPV is not necessary unless antibody titer is below detectable levels, in which case revaccination is recommended prior to chemotherapy (Arrowood & Hayney, 2002). Due to the loss of immunity to poliovirus, post-HSCT patients should receive a series of three IPV injections beginning 6 months posttransplant at monthly intervals plus a booster dose at 18 months post-HSCT. Only the IPV formulation should be used in post-HSCT patients, as opposed to the live formulation (OPV). Oral polio vaccine should be avoided due to the risk of vaccine-associated paralytic episodes occurring in immunocompromised patients (Pirofski & Casadevall, 1998; CDC, 2012).

Adverse events associated with IPV are generally mild. Oral polio vaccine is a live vaccine that has been associated with rare but serious vaccine-induced paralytic poliomyelitis in healthy recipients. Hypersensitivity is a side effect for both formulations as it contains traces of bacitracin (OPV only), streptomycin, and neomycin (both OPV and IPV). The IPV formulation is the polio vaccine of choice for most patients.

## Meningococcal Vaccine

*Neisseria meningitides* is a bacterial infection that affects the brain and spinal cord. The consequences of such an infection could lead to meningitis, an inflammation of the meninges surrounding the central nervous system, which can be life-threatening. Most often, patients will present with a series of symptoms that include but are not limited to headache, neck stiffness, fever, altered mental status, and vomiting. Due to the seriousness of the condition, the use of vaccine as prophylaxis may play a role in preventing such a deleterious condition while ensuring long-term protective effects.

There are currently two formulations of meningococcal vaccine available: meningococcal conjugate quadrivalent vaccine (MCV4) and meningococcal polysaccharide vaccine (MPSV4). MCV4 is administered intramuscularly while MPSV4 is given subcutaneously. Age is a major determinant for immunogenicity. A child will have an overall antibody response that is only 10% of the adult vaccine (Lepow, 1994). Therefore, MCV4 is recommended for individuals aged 2 to 55 while MPSV4 is recommended for those over 55. Several studies have demonstrated that similar to the pneumococcal vaccine, the conjugated meningococcal vaccine is more immunogenic than the polysaccharide-based meningococcal vaccine (CDC, 2012).

Meningococcal vaccines are recommended in many nononcologic conditions, such as for patients with anatomic or functional asplenia and for college freshmen living in dormitories. The meningococcal vaccines also play an important role for oncologic patients as well as for patients receiving eculizumab (Soliris), per the package insert recommendation (Alexion, 2011).

In pediatric patients, meningococcal vaccine produced variable responses among acute leukemics and suggested the likelihood of response based on proximity to chemotherapy (Yu et al., 2007). Patients with hematologic or oncologic malignancies (particularly Hodgkin disease) who are at high risk of infection should receive the meningococcal vaccine at least 1 week prior to starting therapy. Revaccination is recommended 5 years after the completion of therapy for Hodgkin disease, and then every 5 years after that (Ambrosino & Molrine, 1993). In post-HSCT patients, vaccination with the conjugated meningococcal vaccine starting 6 to 12 months posttransplant may be considered if the likelihood of contracting Neisseria meningitides is high.

Common adverse events associated with the meningococcal vaccine are generally mild, including fever and pain at the injection site. Other rare but serious adverse events may include burning, numbness, and breathing difficulty.

## Human Papillomavirus Vaccine

Human papillomavirus (HPV) is a sexually transmitted infection that has been associated with the development of genital warts, cervical cancer, and anal cancer. In 2009, a three-dose vaccine was developed to protect against the four most common strains of HPV leading to cervical cancer (types 6, 11, 16, and 18). Currently, the HPV vaccine is indicated for the prevention of cervical cancer and genital warts in women who are 9 to 26 years old who have not been exposed to the virus. In males, it is approved for the prevention of genital warts and anal cancer in individuals 9 to 26 years old (CDC, 2012). In the oncology population, it is optional, if age appropriate.

The most common adverse events may include pain and swelling at the injection site, headache, fever, nausea, and fainting. While 92% of the reports were deemed noncritical, 8% of the reports were critical in nature (including death, life-threatening illness, and hospitalization). Guillain-Barré syndrome has also been reported after administration of the HPV vaccine.

## Measles-Mumps-Rubella Vaccine

Measles is a viral infection that affects the respiratory system. Mumps is a viral condition inducing inflammation of the salivary gland and the testicles. Rubella, also known as German measles, is a viral infection that presents as a rash on the face, trunk, and limbs. In some cases, it can also cause joint pain, swollen glands, and conjunctivitis. Together, all three highly contagious conditions have led to widespread outbreaks causing hundreds of thousands of deaths. In the 1960s, the introduction of a measles vaccine diminished the number of reported cases each year. In 2000, the United States declared the elimination of measles. Rubella and mumps cases also sharply declined following the introduction of their respective vaccines. In general, when live vaccines are given individually, they must be given on three separate occasions. Since the introduction of the combined measles, mumps, and rubella (MMR) vaccine, individuals can receive the three vaccines as a single injection, inducing quicker immunity while minimizing pain (CDC, 2012).

The MMR vaccine is given as two separate subcutaneous injections at least 1 month apart to produce long-lasting immunity. In general, the MMR vaccine is included in the childhood vaccination schedule before the age of 2. Repeat vaccination is not generally recommended in adults, unless there is evidence of a lack of immunity as determined by a low or absent titer. In patients undergoing chemotherapy, live vaccines should be avoided, especially in immunocompromised patients (Ljungman et al., 1989; Kroger et al., 2011). In order to ensure protection to individuals undergoing chemotherapy, household members and close contacts should be immunized with MMR. Although the evidence behind the use of live MMR vaccine in cancer patients is not strong, the CDC’s Advisory Committee on Immunization Practices suggests the use of MMR vaccination for leukemic patients in remission no sooner than 3 to 6 months after completion of all chemotherapy treatment (CDC, 1998; Arrowood & Hayney, 2002).

Unlike patients who receive chemotherapy without stem cell transplant, post-HSCT patients will experience in a decrease in and eventually a loss of MMR immunity from most previous vaccination within the first few months after transplant. Although vaccination with live attenuated MMR vaccine is generally not recommended immediately post-HSCT, it should be reconsidered at least 2 years posttransplant. Patients should be free from chronic GVHD and off immunosuppression at the time of revaccination (Ljungman et al., 1989; Kroger et al., 2011).

The common adverse reactions to MMR vaccine may include fever, malaise, and rash up to 3 weeks postvaccination. Joint pain may be more evident in elderly women. In rare instances, anaphylaxis has been reported. Rare neurologic disorders have also been reported.

## Varicella Vaccine

Chickenpox is a highly contagious condition induced by the varicella zoster virus (VZV). It presents as an itchy skin rash, particularly on the body and head. It is spread by direct exposure to the rash secretions. There is generally a lag time of a few days to weeks after the individual has been exposed before the rash presents. Chickenpox appearing late in life is a reactivation of the VZV, otherwise known as shingles. In addition to proper hygiene, vaccination plays a role in providing protection (CDC, 2012).

The varicella vaccine, introduced in the 1990s, is a live vaccine that provides protection against chickenpox and herpes zoster. It is generally recommended for children under 13, with the first given dose at 12 to 15 months and a repeat dose given at 4 to 6 years. For individuals who are older than 13 but have never been introduced to chickenpox, the two doses should be administered 4 to 8 weeks apart. In patients undergoing chemotherapy, live varicella vaccine is not recommended, especially in immunocompromised patients (CDC, 2012). In order to ensure protection to individuals undergoing chemotherapy, household members and close contacts should be immunized with varicella vaccine (Marin, Güris, Chaves, Schmid, & Seward, 2007).

Similar to the loss of MMR immunity, post-HSCT patients will undergo a decrease in and eventually a loss of immunity to varicella. Although vaccination with live attenuated varicella vaccine is generally not recommended immediately post-HSCT, it should be reconsidered at least 2 years posttransplant. Patients should be free from chronic GVHD and off immunosuppression at the time of revaccination (Ljungman et al., 1989; Kroger et al., 2011).

Common side effects may include soreness and redness at the injection site, fever, and rash. Seizures, pneumonia, and anaphylaxis are rare but serious side effects that have been reported.

## Live Vaccines

In general, live vaccines (MMR, varicella, and intranasal influenza) should be avoided in patients undergoing concurrent chemotherapy and posttransplant patients. The timing and method of protection differs between these two populations.

Live vaccines should be withheld in patients who are prior to or receiving concurrent chemotherapy. Instead, it is recommended that household members and close contacts be fully immunized to lessen the likelihood of the infection. Patients who are immunosuppressed, including posttransplant patients, should wait at least 24 months posttransplant and until they are no longer receiving immunosuppression, free from graft-vs.-host disease, and have immunologic response before receiving live vaccines. As previously discussed, due to the dramatic decrease and eventually a complete loss of immunity from previous vaccinations, live vaccines may be reconsidered when the above criteria are met in order to reinitiate lifelong protection after transplant (Ljungman et al., 2009; Hilgendorf et al., 2011).

## Donor Vaccination

Although there are no specific recommendations, donor vaccination with Td, PCV7, and Hib has been associated with an improvement in posttransplant immunity for the recipient of the stem cell transplant (Ljungman et al., 2009). In addition, early administration of the hepatitis B vaccine posttransplant may assist with the transfer of immunity from the donor to the recipient of the transplant, if the donor is immunized prior to stem cell collection (Ljungman et al., 2005; Hilgendorf et al., 2011).

## Conclusion

In most instances, patients undergoing chemotherapy should receive both influenza and pneumococcal vaccine at least 2 weeks before starting chemotherapy with potential additional vaccines based on the clinical picture (see Table 6). If possible, inactivated vaccines should be administered at a minimum of 2 weeks prior to starting therapy. If the previous scenario is not possible, then one should consider receiving vaccination in between cycles. Vaccinating too close to chemotherapy could lead to the loss of durable immunity (Hilgendorf et al., 2011; Ljungman et al., 2009). In most cases, many post-HSCT patients are viewed as "never vaccinated." These individuals are recommended to be revaccinated with certain vaccines, while some are optional and others should be avoided (see Table 6).

**Table 6:**
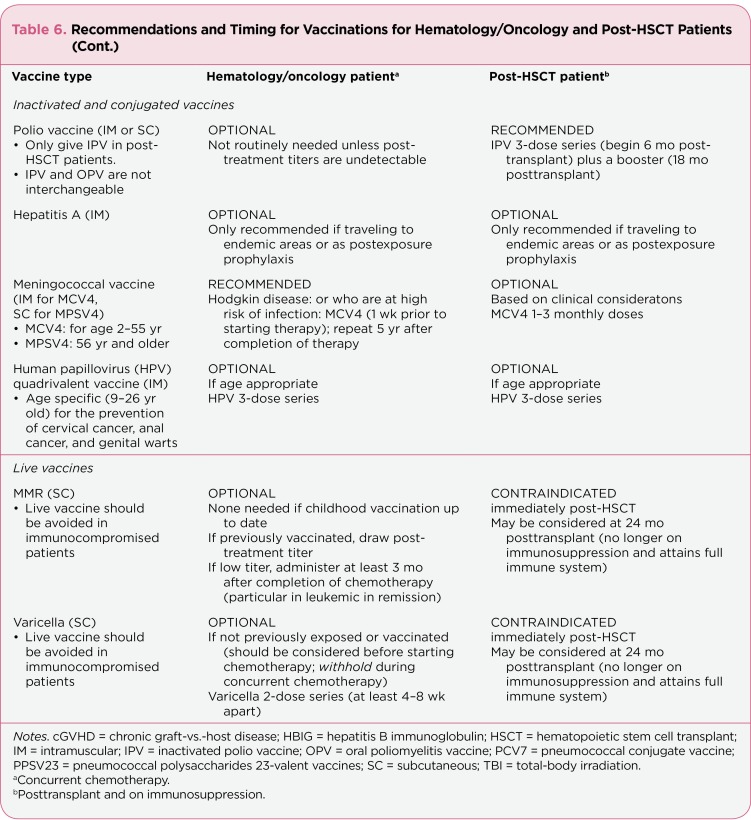
Table 6. Recommendations and Timing for Vaccinations for Hematology/Oncology and Post-HSCT Patients

**Table 6:**
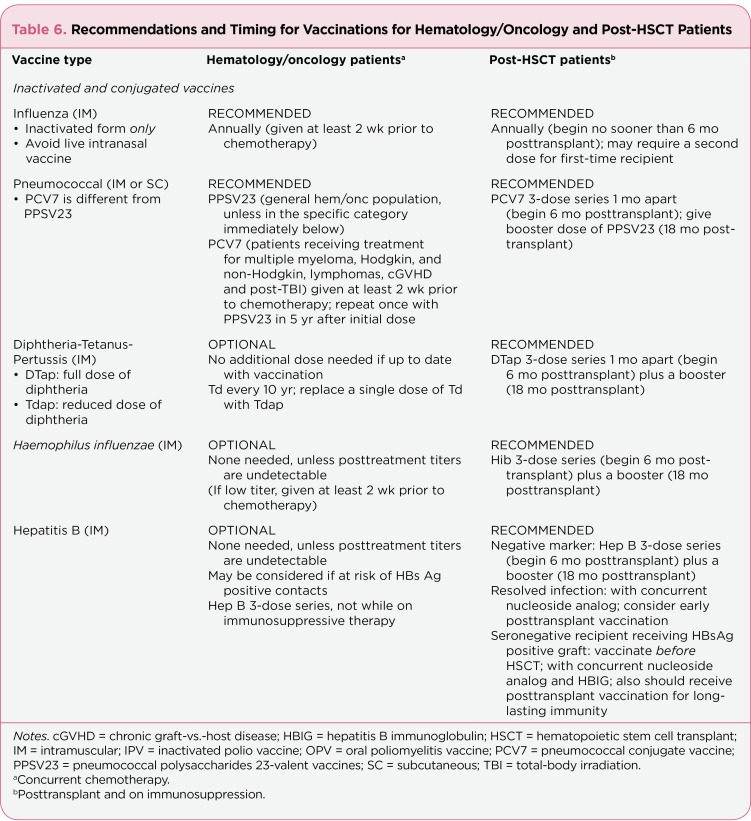
Table 6. Recommendations and Timing for Vaccinations for Hematology/Oncology and Post-HSCT Patients (Cont.)

For the general population, childhood vaccination has led to the elimination of many preventable diseases. Likewise, the same approach in the hematology and oncology population has been beneficial. In the immunosuppressed population, appropriate timing and avoidance of live vaccines will not only provide protection against these diseases but also confer longer-lasting immunity. Advanced practitioners can benefit from recognizing the importance and implications of each vaccine for the hematology, oncology, and posttransplant populations.
